# Altered connectivity of the right anterior insula drives the pain connectome changes in chronic knee osteoarthritis

**DOI:** 10.1097/j.pain.0000000000001209

**Published:** 2018-03-16

**Authors:** William J. Cottam, Sarina J. Iwabuchi, Marianne M. Drabek, Diane Reckziegel, Dorothee P. Auer

**Affiliations:** a Arthritis Research UK Pain Centre, University of Nottingham, Nottingham, United Kingdom; b NIHR Nottingham Biomedical Research Centre, Queen's Medical Centre, University of Nottingham, Nottingham, United Kingdom; c Sir Peter Mansfield Imaging Centre, School of Medicine, University of Nottingham, Nottingham, United Kingdom

**Keywords:** Chronic pain, Resting-state, Knee osteoarthritis, Neuroimaging, Functional connectivity

## Abstract

Resting-state functional connectivity (FC) has proven a powerful approach to understand the neural underpinnings of chronic pain, reporting altered connectivity in 3 main networks: the default mode network (DMN), central executive network, and the salience network (SN). The interrelation and possible mechanisms of these changes are less well understood in chronic pain. Based on emerging evidence of its role to drive switches between network states, the right anterior insula (rAI, an SN hub) may play a dominant role in network connectivity changes underpinning chronic pain. To test this hypothesis, we used seed-based resting-state FC analysis including dynamic and effective connectivity metrics in 25 people with chronic osteoarthritis (OA) pain and 19 matched healthy volunteers. Compared with controls, participants with painful knee OA presented with increased anticorrelation between the rAI (SN) and DMN regions. Also, the left dorsal prefrontal cortex (central executive network hub) showed more negative FC with the right temporal gyrus. Granger causality analysis revealed increased negative influence of the rAI on the posterior cingulate (DMN) in patients with OA in line with the observed enhanced anticorrelation. Moreover, dynamic FC was lower in the DMN of patients and thus more similar to temporal dynamics of the SN. Together, these findings evidence a widespread network disruption in patients with persistent OA pain and point toward a driving role of the rAI.

## Abstract 2

Supplemental Digital Content is Available in the Text.

A resting-state functional magnetic resonance imaging study of chronic knee osteoarthritis pain using a range of functional connectivity analyses to better understand brain network changes.

## 1. Introduction

Attention to the external world is driven by the salience of perceived stimuli, which is particularly relevant for threat and pain stimuli. There is a mutual link between, and interdependence of, perception and attention to pain known from everyday life experience and demonstrated at the neural level.^59^ A brain network implicated in this process is the “salience network” (SN).^50^ The core SN is formed by the bilateral anterior insula and the anterior middle cingulate cortex supporting the integration of external information, previous experience, and concurrent homeostatic state, to orient attention.^39^ Therefore, the SN is expected to play a dominant role in chronic pain, a condition believed to represent a maladaptive state of heightened arousal and pain anticipation. Chronic pain is also linked to aversive learning in which the sufferer might continually reevaluate the salient value of pain.^3,9^

Chronic pain is not consistently defined, but there is consensus that any criteria should reflect pain that lasts beyond the normal tissue healing time. It may be caused by any number of underlying pathologies such as arthritis, neuropathy, or migraine and is very common affecting between 33% and 50% of adults in the United Kingdom.^19^ The cause is not fully understood but central mechanisms have been implicated as predisposing factors.^57^ Interestingly, studies focusing on brain responses to evoked pain failed to consistently distinguish patients from healthy volunteers.^45,55^ By contrast, resting-state functional magnetic resonance imaging (fMRI) studies regularly report altered functional connectivity (FC) in the default mode network (DMN), believed to relate to interoception and mind wandering, in patients with chronic pain. Default mode network changes in chronic pain were mainly reported as increased connectivity,^4–6,10,11,24,35,37,41^ whereas others find the opposite.^6,24^ The most common region showing increased FC with the DMN in chronic pain is the insula^4,6,10,11,37,41,43^ suggesting increased binding between SN and DMN. Previous work in healthy volunteers has demonstrated the right anterior insula (rAI) as the main causal output within the SN, projecting to and influencing the central executive network (CEN) and DMN acting as a key switch between these networks.^22,39,53^ There are, however, surprisingly few studies reporting SN changes in chronic pain with 2 studies reporting increased connectivity,^7,24^ and 2 with no difference compared with controls.^6,27^ To make sense of these reported differences and discrepancies between studies, a broader approach combining different measurements of FC after rigorous quality control of putative confounds in chronic pain is still lacking.^47^

Given the importance of the SN in orienting attention and the primacy of the rAI, we hypothesised that chronic pain is characterised by altered rAI FC that causally alters depending network function (eg, the DMN). To test this, we used seed-to-brain connectivity analysis to assess resting-state networks in subjects with chronic knee osteoarthritis (OA) pain compared with controls. To provide better context for findings, comprehensive analysis was then undertaken to assess internetwork connectivity, effective and dynamic connectivity changes, and their interrelation with pain characteristics, structural and cerebral blood flow (CBF) data using available multimodal data from this cohort.^15,17^ This in-depth analysis will allow for a better mechanistic understanding of reported outcomes than previous reports.

## 2. Methods

### 2.1. Participants

Thirty-nine patients with radiographically defined chronic knee OA (15 men; median age 65 years; range 48-84 years; range of pain duration 1-38 years) and 25 healthy subjects (8 men; median age 65.5 years; range 51-80 years) were included. None of the subjects had a past or current diagnosis of any psychiatric or major neurological illness. Participants were recruited from orthopaedic clinics and from those who had participated in previous research and had given consent for further contact.

The study was run in accordance with the Declaration of Helsinki, and ethical approval was given by the Nottingham Research Ethics Committee 2 (Ref: 10/H0408/115). Signed and informed consent was obtained from all participants before enrolling in the study.

### 2.2. Demographic and questionnaire data

Directly before the scan session, all subjects undertook a battery of psychometric questionnaires including measures of educational level, low mood (Beck's Depression Index), anxiety (State-Trait Anxiety Index), pain state (McGill Pain Questionnaire), and pain catastrophizing (Pain Catastrophizing Scale).^8,18,38,52,54^ Patients were also asked to rate their knee pain using a visual analogue scale (0-100; 0 meaning no pain and 100 worst imaginable pain).

### 2.3. Magnetic resonance imaging data acquisition

All subjects underwent multimodal MRI at 3T (Discovery MR750; GE Healthcare) using a 32-channel head coil. BOLD fMRI resting-state data is reported here alongside a T1-weighted anatomical image used for registration purposes. Arterial spin labelling, structural, and magnetic resonance spectroscopy data from this study have been reported elsewhere.^1,15,48^ Resting-state data consisted of 165 single-echo gradient echo-planar volumes acquired over 5 minutes 30 seconds while participants were asked to keep their eyes closed and relax (time to echo/time to recovery [TR] = 32/2000 ms, interleaved acquisition, slice thickness = 3.6 mm, 35 axial slices parallel to anterior–posterior commissure plane, flip angle = 90°, matrix = 64 × 64, field of view = 240 mm, in-plane resolution = 3.75 mm^2^). High-resolution anatomical images were acquired using a gradient echo sequence (time to echo/TR = 3.324/8.492 ms, TI = 450 ms, slice gap = 1 mm, field of view = 256, matrix = 256 × 256, flip angle = 12°, voxel resolution = 1 mm^3^.

### 2.4. Quality control

Functional MRI underwent a 2-step quality checking method (examples can be found in supplemental materials Figure 1, available online at http://links.lww.com/PAIN/A556). First, using an in-house pipeline following criteria outlined in the FBIRN study,^21^ we assessed raw image quality and found a number of data sets with excessive noise in the frequency spectra that were hence excluded. Second, data were excluded if it was of poor quality due to movement (>1 mm). In total, 20 data sets were discarded (patients = 14, controls = 6) reflecting the rigorous quality control. Group demographics after exclusions can be found in Table 1.

**Table 1:**
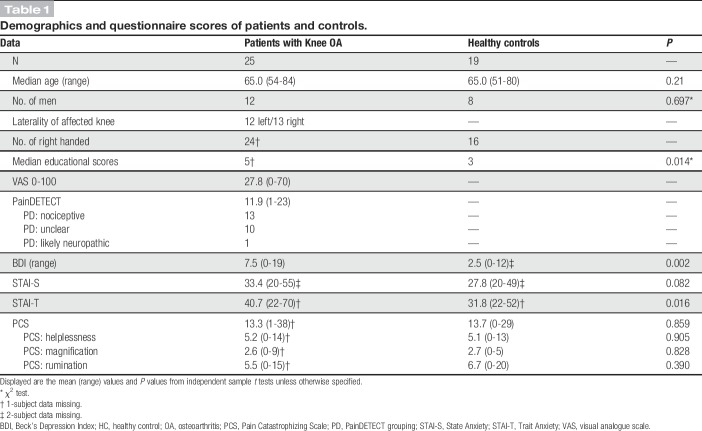
Demographics and questionnaire scores of patients and controls.

### 2.5. Image preprocessing

Image preprocessing was performed using FSL 5.0.8 (FMRIB software library), initially performing preprocessing steps including removal of the first 5 imaging volumes to allow for signal equilibrium effects, high-pass temporal filtering (0.01-Hz cutoff), interleaved slice-timing correction, motion correction,^30^ brain extraction, and spatial smoothing (5-mm full width at half maximum). Linear registration (FLIRT) to the T1-weighted image (BBR) and Montreal Neurological Institute standard space (12° of freedom) was performed.^30,31^ All data sets were denoised using ICA + FIX (independent component analysis followed by FMRIB's ICA-based noise reduction.^23,49^ To additionally control for physiological/scanner-related noise, time series data were extracted for each subject from cerebrospinal fluid (CSF) and white matter (WM). This was achieved using FMRIB's Automated Segmentation Tool (FAST) to calculate tissue segmentations from the subjects' T1-weighted images. The CSF and WM maps were then transformed to fMRI space using the previously calculated transform and were volume thresholded to retain only the top 20 and 198 cm^3^, respectively, to minimise partial volume and global demeaning effects.^12^ Mean CSF and WM time series were then extracted per subject using these masks and regressed out of the data alongside the 6 motion parameters (x, y, z, roll, yaw, and pitch) as part of the subsequent GLM analysis. Global signal regression was not performed.

### 2.6. Resting-state connectivity

**Figure 1.**
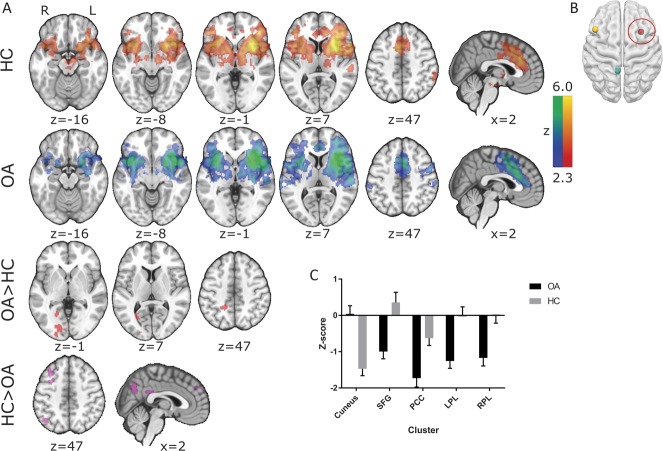
Resting-state connectivity in (A) healthy controls (HCs), patients with chronic knee osteoarthritis (OA) and differences between these groups (FWE *P* < 0.05) of the (B) right anterior insula. (C) Mean (+SEM) z values of those clusters displaying a significant group difference are displayed on a bar graph to visualise the anticorrelations observed in patients with chronic knee OA. All slice images are displayed in radiological convention (right hemisphere is displayed on the left of the figure). PCC, posterior cingulate cortex.

**Figure 2.**
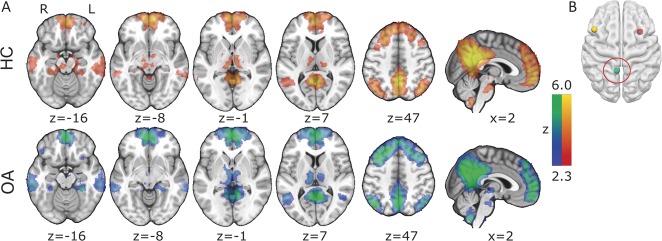
Resting-state connectivity in (A) HCs, patients with chronic knee OA (FWE *P* < 0.05) of the (B) posterior cingulate cortex. All slice images are displayed in radiological convention (right hemisphere is displayed on the left of the figure). HC, healthy control; OA, osteoarthritis.

**Figure 3.**
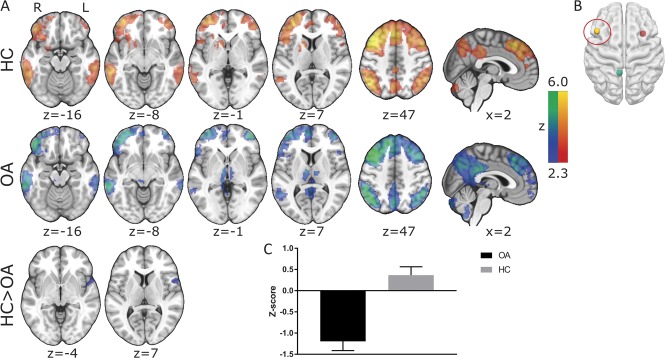
Resting-state connectivity in (A) healthy controls (HCs), patients with chronic knee osteoarthritis (OA) and differences between these groups (FWE *P* < 0.05) of the (B) left dorsolateral prefrontal cortex. (C) Mean (+SEM) z values of those clusters displaying a significant group difference are displayed on a bar graph to visualise the anticorrelations observed in patients with chronic knee OA. All slice images are displayed in radiological convention (right hemisphere is displayed on the left of the figure).

Seed-based connectivity was calculated using 3 regions of interest (ROI) to investigate the 3 networks of interest using the SN—rAI (MNI coordinates [MNIxyz] = 32, 16, 6), CEN—the left dorsolateral prefrontal cortex (lDLPFC; MNI coordinates [MNIxyz] = −46, 20, 44), and the DMN—posterior cingulate cortex (PCC; MNI coordinates [MNIxyz] = −8, −50, 28) based on previously published coordinates.^24,56^ See Figures 1–3 for visual presentation of the seed regions. All ROIs were spherical with a 6-mm radius.

### 2.7. Internetwork connectivity

Measures of FC between network hubs were calculated by means of (1) correlative connectivity and (2) Granger causality measures. Correlative connectivity was assessed using the 3 extracted time series for the rAI, lDLPFC, and PCC. Pearson correlation coefficients were calculated between these 3 time series per subject and then the Fisher *r*-to-*z* transformation was applied.

Bivariate first-order coefficient-based voxel-wise Granger causality analysis (GCA) was calculated using the REST fMRI toolbox.^51^ Granger causality analysis calculates an estimate of the causal effect of a region of interest on each voxel in the brain (X-Y effect) and the reverse (Y-X effect). A positive coefficient from X to Y signifies that activity in the region of interest exerts a positive influence on the activity of a specific region. Conversely, a negative coefficient from X to Y indicates that activity in the region of interest (X) exerts a negative influence on the activity of a specific region (Y). Seed-to-whole-brain Granger scores were calculated for each of the 3 ROIs per subject. For each seed region, mean Z values were extracted from the remaining 2 ROIs from the X to Y contrast only.

### 2.8. Dynamic functional connectivity

To assess intranetwork variability, a sliding-window analysis was performed to calculate SD using an in-house Matlab code. The 3 networks were defined by performing group ICA analysis (multisession temporal concatenation) on all 44 subjects through FSL Melodic (v3.14) limited to 25 components. Default mode network, CEN, and SN components were then visually assessed (see supplementary Figure 2, available online at http://links.lww.com/PAIN/A556). These components were then thresholded at Z = <2.3 and back registered into subject space to use as masks in the sliding-window analysis. Intranetwork analysis was performed by extracting time series using singular value decomposition (to reduce the effect of outlier values on the extracted time series) from each individual cluster within a network (see supplementary Table 1 for cluster details, available online at http://links.lww.com/PAIN/A556). A window of 100 seconds or 50 TRs was used as this corresponds to the frequency of 0.01 Hz—the lowest frequency of interest. Pearson correlation coefficients were then calculated per window and underwent Fisher's *r*-to-*z* transformation. The SD of these correlation values over all windows was calculated per connection per subject. To have a single metric per network, the mean SD was calculated across all connections within a network. These mean SD values were subsequently taken into further analysis such that each subject had a single value per network. In addition, for clarity, a table of SD for each network over window sizes of 20 to 50 is supplied in the supplementary analyses to show the consistency of the results regardless of the window size used (supplementary Figure 3, available online at http://links.lww.com/PAIN/A556).

### 2.9. Multimodal data

To further characterise changes observed from these 3 seed regions, CBF and gray matter densities from participants (T1 data were available for all subjects, but CBF data were unavailable from 4 subjects [OA = 22, healthy control [HC] = 18]) were extracted. Cerebral blood flow data were taken from previously published material whereas gray matter density was calculated by using the FSL VBM toolbox.^15,17^ Gray matter density was then extracted by back registering the seeds into subject space.

### 2.10. Statistical tests

Demographic data (age, sex), questionnaire scores (Beck's Depression Index, Pain Catastrophizing Scale, State-Trait Anxiety Index), regional CBF, gray matter density scores, and levels of mean relative motion recorded during the fMRI scan were compared between groups using independent sample *t* tests in Minitab 17.2.1. Measures of internetwork connectivity were also compared using 2-sample *t* tests within Minitab 17.2.1. Significance for these tests was set at *P* < 0.05.

For subject-level image analyses, time series from each of the 3 ROIs was used as predictors in individual regression models including 8 nuisance covariates (CSF, WM, and 6 motion parameters) using FSL FEAT. Group-level analyses were additionally controlled for mean relative motion,^25^ and statistical analyses were performed using FLAME 1 (a mixed-effect general linear model within FSL; FWE corrected Z > 2.3, cluster significance *P* < 0.05) comparing connectivity in patients with OA vs HCs. Repeat analyses additionally controlling for age were performed to rule out age as a confound on the reported connectivity.^2^

Relationships between regions where FC was found to be significantly different between patients and controls, and seed-based scores of gray matter density and CBF were investigated in Minitab 17.2.1 using stepwise linear regression. *Z*-scores from the brain regions were entered as dependent variables, whereas all other reported measures (pain at time of scanning, McGill Pain Questionnaire subscores; sensory, affective, and evaluative), neuropathic-like symptoms, anxiety, low mood, and catastrophizing subscales were entered as independent variables in the model. Holms-Bonferroni multiple-test correction was used for these regression analyses.

## 3. Results

### 3.1. Demographics

No significant group differences were found in age, sex, catastrophizing, or mean relative motion during the fMRI scan (*P* = 0.36). Patients with OA had lower educational levels (reflected by higher scores), lower mood, and higher trait anxiety scores than HCs (Table 1).

### 3.2. Functional connectivity of the salience network, default mode network, and central executive network

Seed-based FC of the rAI showed the core SN consisting of the bilateral anterior insula and the anterior middle cingulate cortex with further clusters within the bilateral putamen, caudate heads, frontal poles, right amygdala, and temporoparietal junction. Patients additionally displayed functional rAI connectivity with the left temporoparietal junction and the right precentral gyrus (Fig. 1A). Group comparison revealed a cluster of increased rAI FC within the cuneus in patients (FWE *P* < 0.05, Fig. 1A and Table 2) and several clusters of decreased rAI FC in patients compared with controls in areas associated with the DMN including the PCC, bilateral parietal areas, and the superior frontal gyrus (FWE *P* < 0.05, Fig. 1A and Table 2). To characterise the nature of relative FC changes between patients and controls, mean *z*-scores were extracted from significant clusters. This revealed that patients exhibited enhanced anticorrelation (negative FC) rather than less positive FC. By contrast, the increased rAI-cuneus FC in patients was linked to an abolished anticorrelation seen in HCs (see Fig. 1C for graphical visualisation).

**Table 2:**
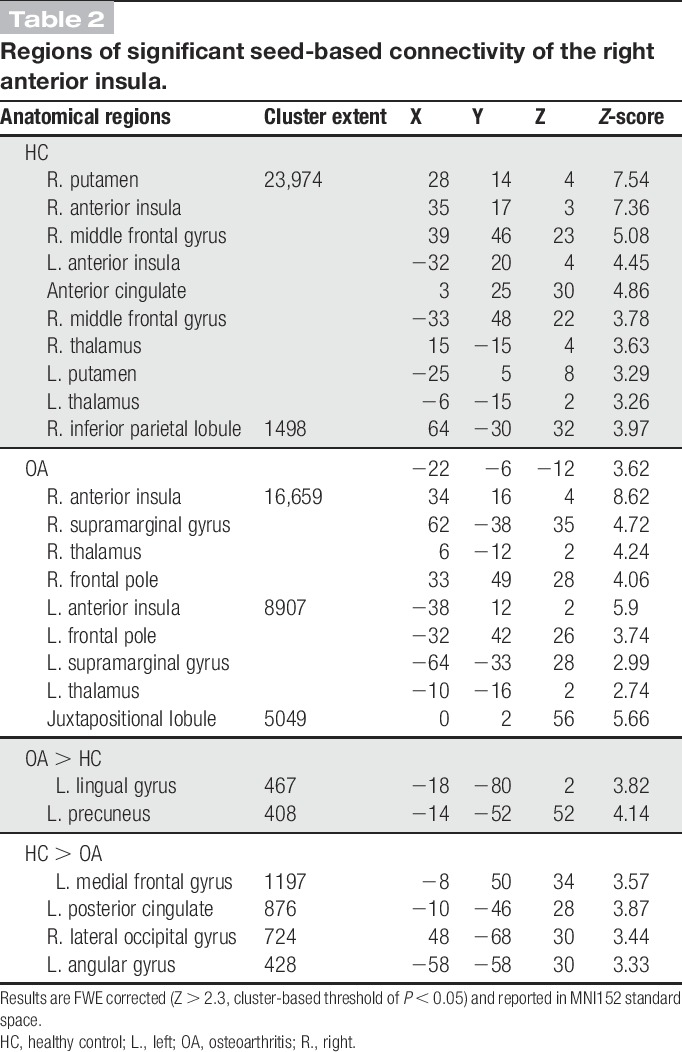
Regions of significant seed-based connectivity of the right anterior insula.

Functional connectivity of the PCC displayed a similar extent of the DMN across both subject groups including the PCC/precuneus, the medial prefrontal cortex, the lateral parietal regions, the bilateral thalami, hippocampi, and the lateral frontal cortex (Fig. 2 and Table 3). Comparison between patients and healthy control groups revealed no significant differences.

**Table 3:**
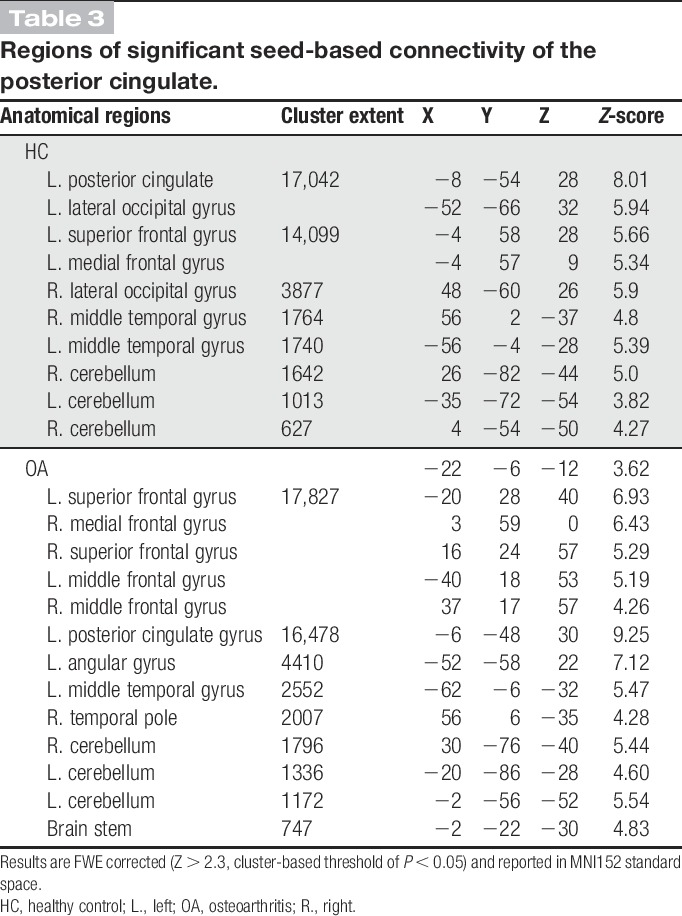
Regions of significant seed-based connectivity of the posterior cingulate.

Functional connectivity of the lDLPFC demonstrated the core CEN including the bilateral DLPFC and posterior parietal cortices. The clusters within the bilateral DLPFC extended into the medial prefrontal regions, whereas additional clusters were also observed in the PCC, the bilateral middle temporal gyri, cerebellum, and the bilateral thalami. Compared with HCs, patients showed reduced CEN FC in a single cluster (FWE *P* < 0.05) that extended superiorly from the right temporal pole into the inferior frontal gyrus (Fig. 3 and Table 4). This was again a finding of increased anticorrelation within the OA cohort, whereas the HC FC was centred just above 0 (see Fig. 3C for visual representation). All seed-based FC results were replicated when additionally controlling for age, thus confirming that this was not a confounding factor in the reported results.

**Table 4:**
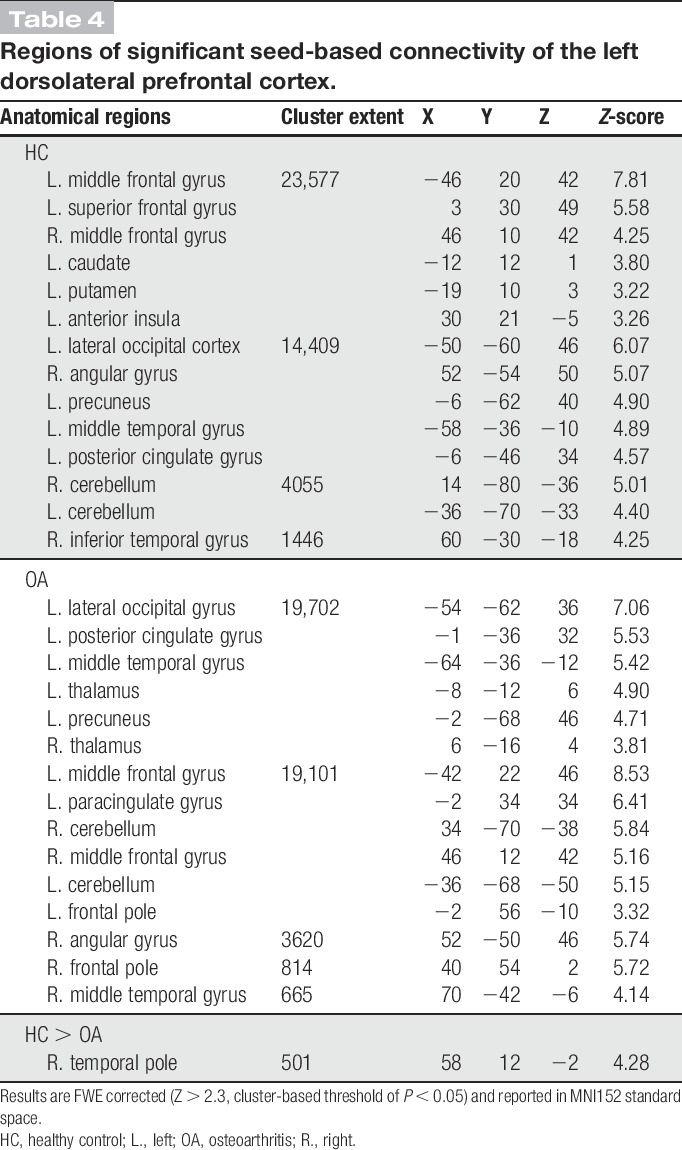
Regions of significant seed-based connectivity of the left dorsolateral prefrontal cortex.

### 3.3. Between-network hub functional and effective connectivity

Seed-to-seed FC, estimated using Pearson correlation coefficients, revealed no significant differences (after multiple-test correction) between patient and healthy control cohorts in connectivity between the pairs of network hubs: rAI-PCC (*t*(41) = −0.06, *P* = 0.95), rAI-lDLPFC (*t*(41) = 0.11, *P* = 0.92), or the PCC-lDLPFC (*t*(41) = 1.53, *P* = 0.13).

By contrast, estimates of effective connectivity derived from Granger causality analysis between these network hubs revealed a significant difference between patient and control groups for the influence rAI exerts (->) on the PCC. We found that rAI had a stronger negative effect on PCC in patients with OA (*t*(33) = −2.26, *P* = 0.03). No other calculated Granger coefficient was found to be significantly different between groups: rAI->lDLPFC (*t*(33) = −1.57, *P* = 0.13), lDLPFC->rAI (*t*(33) = −1.31, *P* = 0.20), lDLPFC->PCC (*t*(33) = −0.65, *P* = 0.52), and PCC->rAI (*t*(33) = −1.40, *P* = 0.17) with a trend towards increased positive influence from PCC->lDLPFC (*t*(33) = 1.89, *P* = 0.07) in patients (see supplementary Figure 4 for graphical representation, available online at http://links.lww.com/PAIN/A556).

### 3.4. Dynamic connectivity

**Figure 4.**
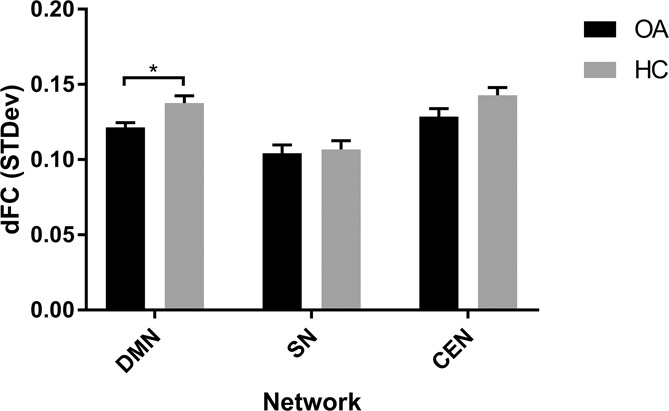
A bar graph displaying the group mean (+SEM) of calculated dynamic functional connectivity over a sliding window of 20 TRs (40 seconds). CEN, central executive network; dFC, dynamic functional connectivity; DMN, default mode network; HC, healthy control; OA, osteoarthritis; SN, salience network.

Intranetwork connectivity also displayed significantly reduced variability within the DMN in patients compared with HCs (*t*(42) = 2.873, *P* = 0.006, Fig. 4). Salience network and CEN networks displayed no significant differences between patients and controls. Post hoc assessment of different sliding-window sizes found that these findings were consistent across sizes from 20 to 50 TRs (supplementary Figure 3, available online at http://links.lww.com/PAIN/A556).

### 3.5. Associations with pain characteristics and anxiety

Post hoc analyses of the rAI FC within those clusters found to be significantly abnormal in OA revealed 2 associations between reported pain and psychological pain scores in patients. First, rAI FC with the PCC and superior frontal gyrus were found to be positively correlated with reported pain intensities (*t*(22) = 2.68, *P* = 0.015 and *t*(22) = 2.1, *P* = 0.048 respectively). Second, we found a negative correlation between lDLPFC FC with the right temporal gyrus and trait anxiety (*t*(22) = −2.26, *P* = 0.035). However, none of the associations survived multiple-test correction.

### 3.6. Are functional connectivity changes linked to changes in brain activation state or atrophy?

The multimodal data set further allowed us to investigate whether the observed FC and eFC changes are simply a reflection of an altered brain state (because of on-going pain) or reflective of brain morphometric alterations previously reported in chronic pain. Hence, we investigated the local CBF averaged from each of the 3 network hub ROIs hypothesising that brain state–related FC changes would be in the same direction as CBF group differences. We did not see any differences in CBF in the investigated network hubs between patients with OA vs controls.

Next, we studied whether FC changes were predicted by GM intensity alterations as have been repeatedly albeit inconsistently found in OA and other chronic pain cohorts. Again, we found no significant correlations between regional GM densities and any of the reported FC metrics suggesting that alterations of the pain connectome are unlikely a simple reflection of on-going pain during the MRI examination nor the result of structural neuroplastic or atrophic changes.

## 4. Discussion

In this study, we undertook a resting-state connectivity analysis of the static and dynamic pain connectome to investigate a possible key role of the rAI in chronic pain. We found increased anticorrelations between the SN and DMN and between the CEN and the left temporal gyrus in patients with chronic OA knee pain compared with pain-free controls. Importantly, we also found increased negative effective connectivity from the rAI to the PCC suggesting an increased negative influence exerted by the SN on the DMN. Moreover, temporal variability of DMN FC was reduced in patients and thus more alike the lower variability seen in the SN in HCs. Taken together, we highlight widespread static, effective, and dynamic FC changes in chronic OA pain that proved independent of activity state (regional CBF), and these connectome changes seem largely driven by rAI FC alterations.

Our study provides evidence that the rAI may drive the altered pain connectome as we found selective effective connectivity changes with an enhanced negative influence of the rAI on the DMN. Patients displayed an increased negative influence of the rAI->PCC, which offers a plausible mechanistic explanation for the observed increased negative connectivity found in the seed-based SN network. Previous studies in HCs have evidenced the causal influence of the SN onto the DMN using time lag and Granger causality analyses showing that the rAI displayed the highest number of outflowing causal connections and the lowest number of inflowing causal connections.^22,53^ Further mechanistic evidence was given by a subsequent study using excitatory and inhibitory transcranial magnetic stimulation (TMS) to interrupt these relationships demonstrating that excitation of CEN/SN nodes induced negative between network connectivity with the DMN, whereas inhibitory TMS resulted in disinhibition of the DMN.^13^ Within the behavioural context that altered “salience” may contribute to or from part of the suffering in chronic pain,^9^ it is then plausible that neuroplasticity changes in the SN caused by the repeated salient and/or painful sensations may underpin observed altered intranetwork and internetwork connectivity changes. Expanding on this, it has also been theorised that chronic pain and negative effect form a continuum of aversive learning, and considering the importance of the SN and its known connectivity with limbic regions, it is tempting to speculate that continuous aversive learning may affect the observed changes in the SN.^3^ Such a notion is supported by our preliminary findings of both anxiety and pain severity relating to functional SN and CEN connectivity in patients.

The study of dynamic network changes may be particularly revealing to understand abnormal network architecture characterised by the inability to switch between states. The neurophysiological basis of network dynamics and the best ways to measure them is an emerging area of active research.^26^ The observed reduced temporal variability (indexed as SD) within the DMN but not the SN or CEN in patients with chronic OA is intriguing. Impaired temporal fluctuation of the DMN FC has been reported in a number of diseases including major depression^33^ and bipolar disorders.^42^ Reduced FC variability has been interpreted to reflect impaired switching between brain states characterised by more segregated subnetworks. Behavioural correlation studies found that reduced variability of FC between networks and/or regions is associated with lower performance in behavioural tasks in HCs and with slower processing speed in patients with bipolar disorder compared with controls.^32,42^ In the context of chronic pain, the observed stronger inhibitory influence of the rAI on the DMN may offer an explanation why within DMN switches may be suppressed. The tonic input of chronic pain or aversive state associated with its anticipation conceivably could affect the dynamic ability of the brain to switch between networks—a hypothesis supported by current data showing altered connectivity between and within networks.^9^ Interestingly, healthy individuals whose cognition was more impacted by a pain distractor or reported a reduced ability to disengage from pain-related thoughts displayed reduced variability between brain regions.^14,34,36^ This suggests that impaired dynamic FC may be an additional important hallmark of the chronic pain connectome that may reflect the inability to detach from clinical pain.

We report here that patients with chronic OA pain display significantly more anticorrelation between the rAI and DMN regions suggesting an altered brain state at rest characterised by increased inhibitory effect of the SN on the DMN. This is interesting as previous reports of group differential DMN-insular connectivity are not consistent in their direction. For instance, several studies report increased (more positive) connectivity in patients,^4,10,37,41^ whereas others report decreased positive connectivity or increased anticorrelations.^6,11^ Although differential DMN-insula connectivity is a consistent finding in the pain literature, the different directions suggest additional factors that modulate the direction of the connectivity alteration. One such influence could be reported pain severity, a factor that we found relates to rAI-PCC connectivity (although not significant after multiple-test correction), which is in line with previous studies that also report relationships between pain and DMN-insula connectivity in multiple pain cohorts.^6,41^ Perceived pain severity in general and during scanning may explain the discrepant findings of reduced anticorrelated connectivity of the SN and DMN found in patients with ankylosing spondylitis compared with controls,^24^ and potentially our finding of no difference in DMN connectivity between groups.

In addition to the major findings within the SN, connectivity of the CEN, specifically in a cluster encompassing the right middle temporal gyrus/inferior frontal gyrus, was found to be negative in patients while controls displayed generally positive connectivity. This cluster is placed almost completely within the SN network, further pointing to the importance of the SN network and its altered internetwork connectivity. In addition, connectivity within this cluster correlated with trait anxiety in patients such that subjects with the highest levels of anxiety displayed the highest SN-CEN anticorrelation. This is interesting in the context that anxiety disorders have previously been related to hyperactivity of the AI within the SN network.^46,50^ Thus, given the known influence of the SN on the CEN, it is possible that increased anxiety (a common comorbidity of chronic pain) plays a role in the maladaptation of SN-CEN connectivity.

Default mode network alterations are the most commonly reported network changes in chronic pain, yet we found no such group DMN FC difference.^5,7,10,35,37,41,43^ It is possible that differences could be due to different patient cohort, subgroups (with more severe pathology and pain severity as discussed above), or analytical choices. For instance, several of these studies used ICA-based connectivity methods, and the majority that did perform seed-based connectivity analyses used ROIs based within the medial prefrontal cortex rather than the PCC. In addition, in this study, we opted for conservative quality assurance on the data, which was likely more stringent than in previous reports and may thus have further differentiated findings.

The reported altered connectivity between networks is similar to internetwork effective connectivity changes that have been reported in mental health disorders such as schizophrenia and major depressive disorder.^28,44^ Interestingly, neuromodulatory treatment has been shown to impact on these internetwork connections in mental health disorders and as such may also have therapeutic potential in the context of chronic pain. For instance, recent work has shown in HCs that individually targeted theta-burst TMS (excitatory stimulation) to the DLPFC alters effective connectivity of the anterior insula.^29^ Thus, the reported altered effective connectivity of the rAI presents as a plausible and modifiable target for neuromodulatory treatment trials.

This study had some limitations that reduce the inference on the findings. First, the study is limited by its cross-sectional design and as such only allows for observational findings. Second, the reported visual analogue scale scores found to correlate with connectivity were collected before the scan, and the level of subjective pain may have changed during the scan. Increasing age is known to affect FC^2,16^ and as such an effort was made to minimise the effect of age by (1) testing against an age- and sex-matched cohort and (2) additionally rerunning the main analyses with age as a covariate (and were subsequently replicated). Educational levels were shown to enhance anticorrelation between the DMN and lDLPFC.^20^ As a common observation, our patient cohort had fewer years of education, and we cannot fully exclude that this may have contributed to observed group differences. Nevertheless, despite significantly lower education levels of our pain cohort, we did not find a difference in DMN connectivity between the 2 groups. We also need to acknowledge that medication use may alter FC, but care was taken to exclude subjects on centrally acting pain killers, such as pregabalin, gabapentin, duloxetine, or strong opiates, whereas 2 participants were on cocodamol. Although we cannot fully exclude a confounding effect from analgesic medication on our reported findings, this is highly unlikely as only 6 of 25 patients reported regular paracetamol use as a peripherally acting drug with limited efficacy. Last, temporal sampling rate would ideally be higher than 2 seconds when performing effective connectivity analyses such as those performed in this report. However, recent studies have argued that effective connectivity results from fMRI can be reproduced at much higher resolutions (eg, using MEG) lending weight to our use of such techniques.^40,58^

## 5. Conclusion

We present novel and comprehensive pain connectome analysis in patients with chronic OA pain and propose the rAI as key region driving static and dynamic network connectivity changes. Although complementing theories of altered salience in chronic pain, these findings also present a novel target and network metrics to mechanistically assess efficacy of neuromodulatory treatments.

## Conflict of interest statement

The authors have no conflict of interest to declare.

Arthritis Research UK funded this study (Grant 18769).

## Supplementary Material

SUPPLEMENTARY MATERIAL

## Appendix A. Supplemental digital content

Supplemental digital content associated with this article can be found online at http://links.lww.com/PAIN/A556.
