# Supplementary material for: Altered connectivity of the right anterior insula drives the pain connectome changes in chronic knee osteoarthritis
Source: Pain. 2018 Mar 16;159(5):929–38. doi: 10.1097/j.pain.0000000000001209 (PMC5916486; doi:10.1097/j.pain.0000000000001209)
Supplement: SUPPLEMENTARY MATERIAL [file jop-159-929-s001.docx]

**Supplementary Material**

**for**

**Altered connectivity of the right anterior insula drives the pain connectome signature in chronic knee osteoarthritis**

William J. Cottam, Sarina J. Iwabuchi, Marianne M. Drabek, Diane Reckziegel, Dorothee P. Auer

**fMRI Quality Assurance**

A two-step image quality assurance procedure was followed in this study. Firstly, an in-house script was run on raw data following guidelines recommended by Friedman and Glover [1]. This script creates several outputs per image including: graphs of frequency spectra and mean time series, measures of signal-to-noise and signal-fluctuation-to-noise ratio among others. Importantly for this dataset, although this other data was calculated, frequency spectra was the clearest indicator of poor data quality at this first stage. Examples of good and bad outputs are included in supplementary figure 1. Secondly, after preprocessing via FSL Feat, images were once again checked for image artefacts and for large amounts of relative motion (as calculated by the MCFLIRT tool – part of the FSL toolbox)[2]. We excluded subjects who displayed total relative motion scores above 1mm.


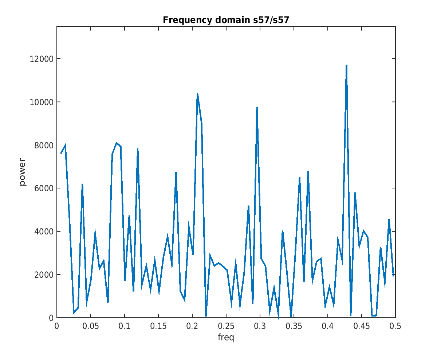

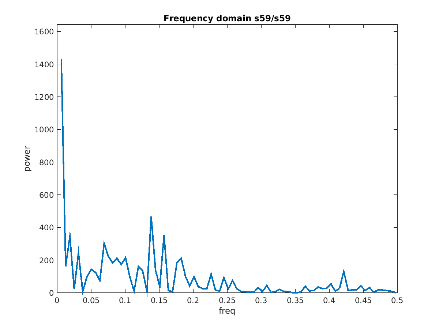

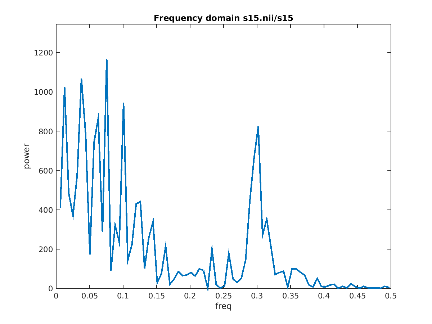

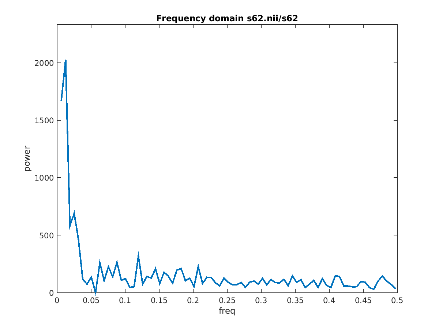


**Included**

**Excluded**

**Supplementary Figure 1.** Examples of frequency spectra calculated using in-house scripts to assess raw fMRI data quality that led to data being included or excluded from further analysis.

**Supplementary Figure 2.** Brain maps depicting the default mode, salience and central executive networks resulting from group independent component analysis. Images are shown in radiographic format (right hemisphere is on the left) and thresholded at Z=>3.0 for visualisation.

**
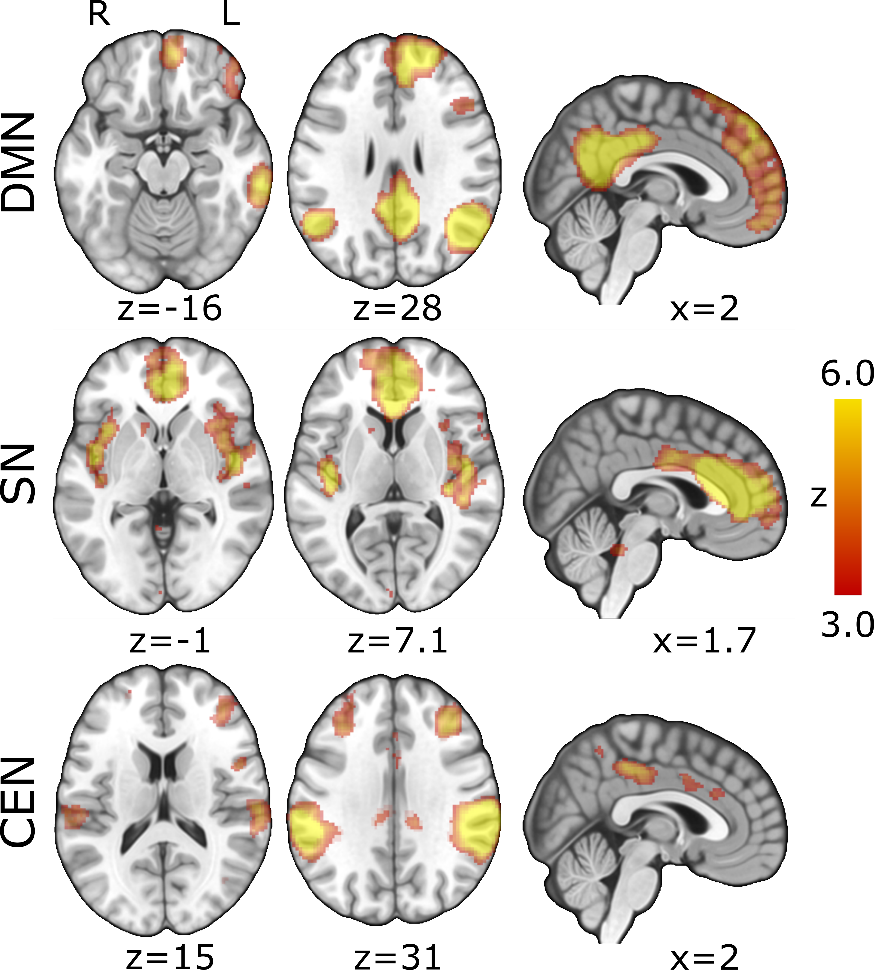
**

**Supplementary Figure 3.** A bar graph displaying the group means (+SEM) of calculated dynamic functional connectivity over sliding windows starting from sizes of 20 TR’s (40 seconds) up to 50 TR’s (100 seconds). Key: dFC – dynamic functional connectivity, STDev – standard deviation, OA – osteoarthritis patients, HC – healthy controls, DMN – default mode network, SN – salience network, CEN – central executive network.


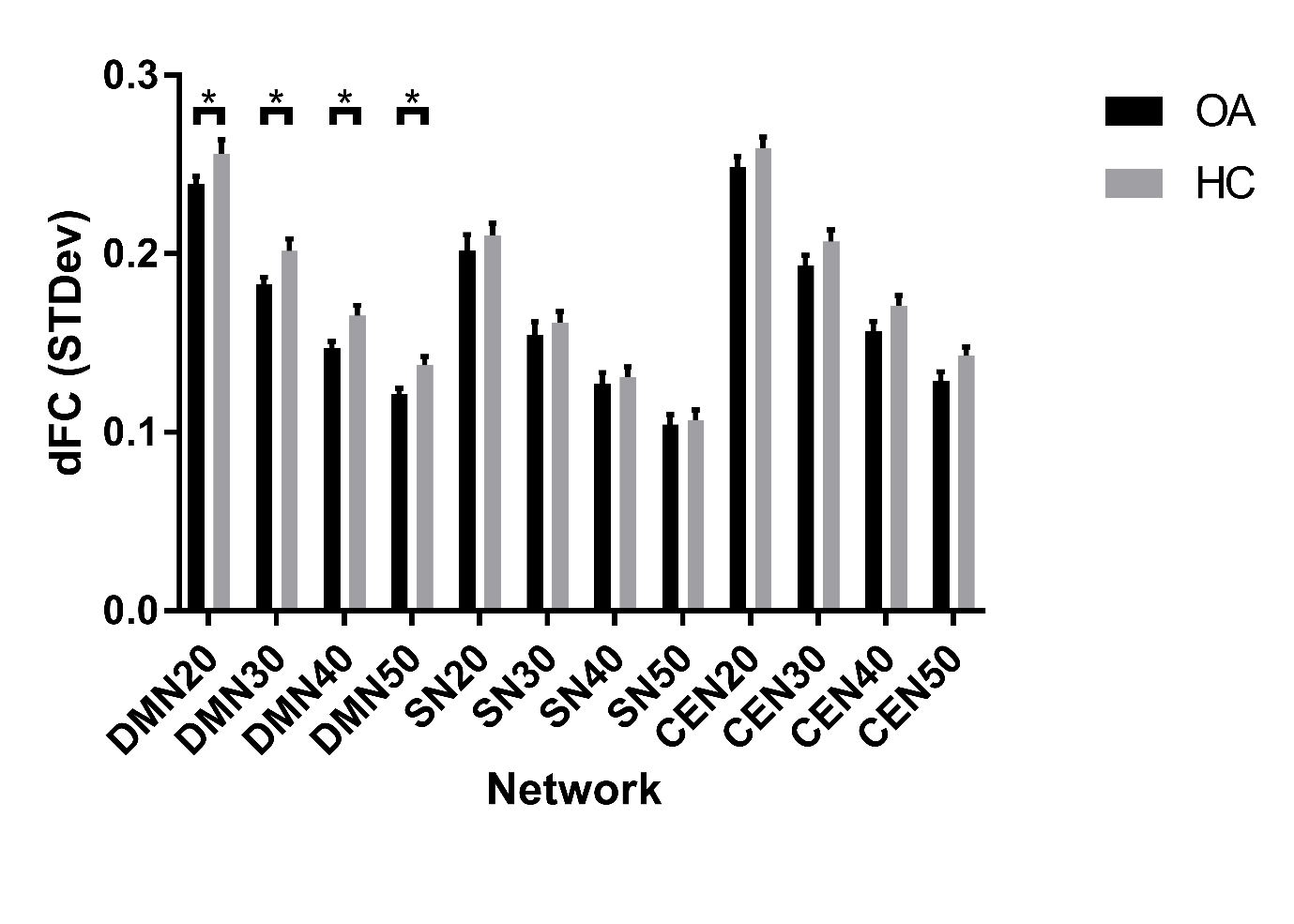


**Supplementary figure 4.** Graphical visualisation of mean (+SEM) group values for calculated seed-to-seed Granger Causality coefficients. Key: OA – osteoarthritis patients, HC – healthy controls, rAI – right anterior insula, PCC – posterior cingulate cortex, lDLPFC – left dorsolateral prefrontal cortex.


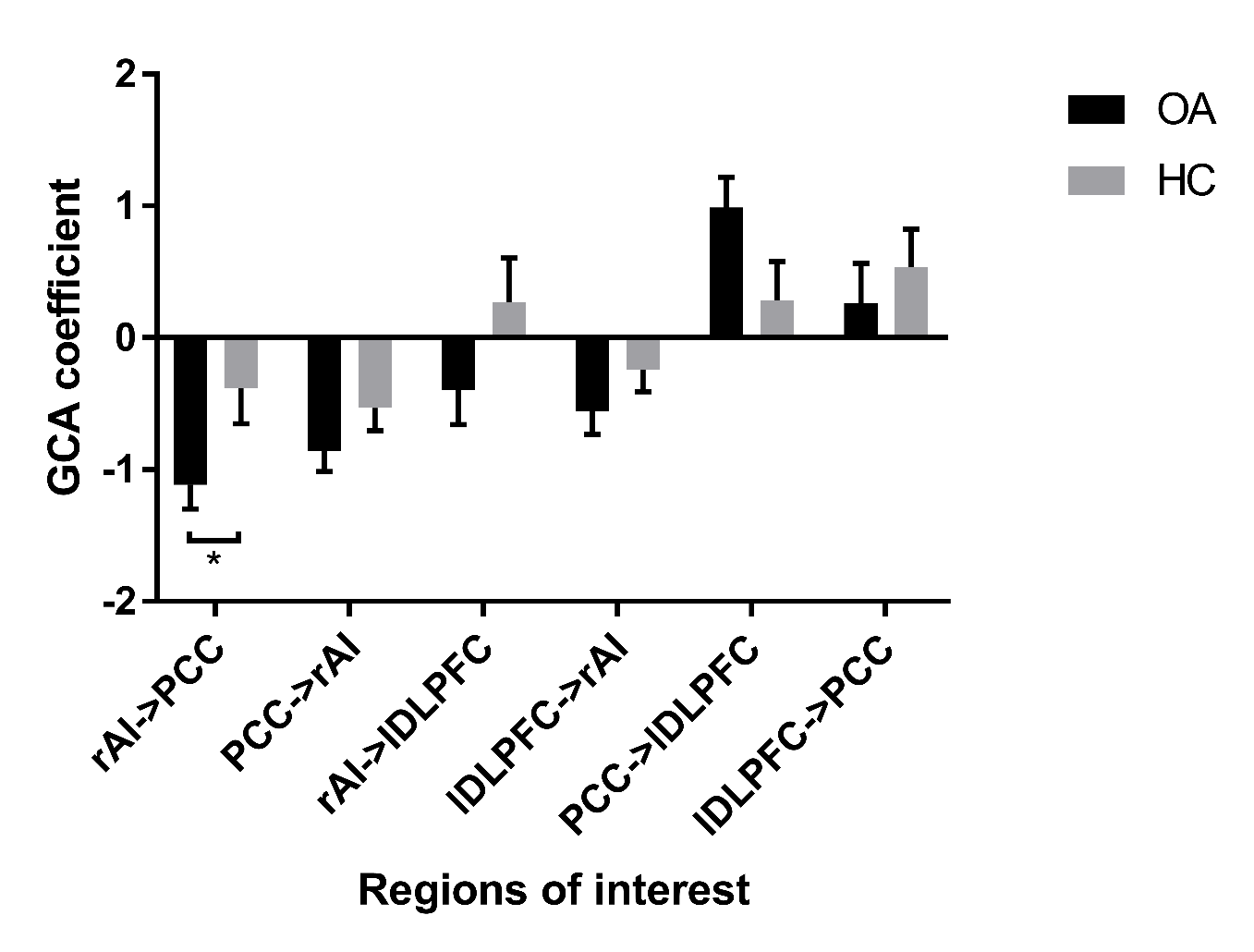


| **Supplementary table 1.**  Significant clusters resulting from ICA analysis of all 44 subjects | | | | | |  |
| --- | --- | --- | --- | --- | --- | --- |
| Anatomical regions | Cluster extent | X | Y | Z | Z-score | |
| **DMN** |  |  |  |  |  | |
| R. Middle frontal gyrus | 6549 | 42 | 16 | 48 | 10.2 | |
| R. Superior frontal gyrus |  | 22 | 34 | 44 | 9.17 | |
| R. Frontal pole |  | 14 | 52 | 32 | 7.58 | |
| R. Paracingulate gyrus |  | 6 | 44 | 26 | 5.86 | |
| R. Lateral occipital cortex | 2094 | 48 | -62 | 36 | 12.7 | |
| R. Posterior cingulate gyrus | 1695 | 6 | -52 | 24 | 10.4 | |
| L. Cerebellum | 639 | -40 | -70 | -46 | 5.72 | |
| L. Angular gyrus | 363 | -50 | -58 | 30 | 6.27 | |
| R. Middle temporal gyrus | 244 | 64 | -32 | -16 | 5.38 | |
| R. Postcentral gyrus | 120 | -44 | -20 | 48 | 4.2 | |
| L. Superior frontal gyrus | 38 | 54 | 80 | 60 | 3.83 | |
| **CEN** |  |  |  |  |  | |
| R. Supramarginal gyrus | 3677 | 60 | -30 | 38 | 10.2 | |
| R. Precuneus |  | 12 | -58 | 58 | 5.88 | |
| R. Planum temporale |  | 64 | -24 | 10 | 3.08 | |
| L. Supramarginal gyrus | 2157 | -58 | -36 | 32 | 9.68 | |
| R. Posterior cingulate gyrus | 447 | 10 | -34 | 42 | 5.38 | |
| R. Middle frontal gyrus | 385 | 36 | 34 | 34 | 5.07 | |
| L. Precuneus | 236 | -10 | -60 | 58 | 4.82 | |
| R. Inferior temporal gyrus | 107 | 54 | -54 | -8 | 4.38 | |
| **SN** |  |  |  |  |  | |
| R. Anterior cingulate gyrus | 3211 | 4 | 38 | 12 | 9.78 | |
| R. Heschl’s gyrus | 468 | 48 | -10 | 4 | 5.98 | |
| L. Heschl’s gyrus | 216 | -42 | -18 | 8 | 5.52 | |
| Brainstem | 5 | 0 | -38 | -26 | 3.27 | |
| Images were thresholded at Z>3.0 and reported in MNI152 standard space. Key: DMN – Default mode network, SN – Salience network, CEN – Central executive network, L. – Left, R. – Right. | | | | | |  |

| Supplementary table 2. | | | | | | | | | | | |
| --- | --- | --- | --- | --- | --- | --- | --- | --- | --- | --- | --- |
| Demographics of osteoarthritis patients | | | | | | | | | | | |
| Participant | Age | Sex | Pain Duration | VAS | BDI | S-Anx | T-Anx | PCS | Mc-S | Mc-A | Mc-E |
| 1 | 54 | f | 38 | 26 | 5 | 15 | 25 | 5 | 6 | 0 | 1 |
| 2 | 59 | f | 25 | 55 | 9 | NA | NA | NA | NA | NA | NA |
| 3 | 59 | f | 3 | 35 | 1 | 16 | 25 | 24 | 10 | 2 | 2 |
| 4 | 71 | f | 14 | 50 | 1 | 10 | 11 | 1 | 12 | 0 | 1 |
| 5 | 70 | f | 10 | 30 | 19 | 22 | 28 | 17 | 22 | 2 | 2 |
| 6 | 81 | f | 30 | 60 | 11 | 13 | 16 | 16 | 11 | 0 | 0 |
| 7 | 72 | m | 12 | 0 | 4 | 10 | 12 | 1 | 0 | 0 | 0 |
| 8 | 63 | f | 20 | 0 | 13 | 18 | 31 | 24 | 12 | 0 | 2 |
| 9 | 56 | m | 10 | 20 | 5 | 13 | 13 | 2 | 10 | 0 | 1 |
| 10 | 67 | m | 10 | 0 | 7 | 15 | 15 | 7 | 10 | 0 | 0 |
| 11 | 80 | m | 10 | 10 | 8 | 10 | 22 | 8 | 11 | 1 | 1 |
| 12 | 84 | m | 2 | 60 | 17 | 15 | 35 | 12 | 14 | 0 | 2 |
| 13 | 57 | m | 10 | 10 | 8 | 21 | 17 | 1 | 7 | 0 | 2 |
| 14 | 63 | m | 9 | 0 | 0 | 17 | 11 | 10 | 0 | 0 | 0 |
| 15 | 61 | m | 1 | 10 | 2 | 10 | 15 | 7 | 6 | 0 | 1 |
| 16 | 63 | m | 5 | 10 | 1 | 10 | 14 | 2 | 4 | 0 | 1 |
| 17 | 65 | f | 6 | 30 | 10 | 15 | 19 | 14 | 1 | 0 | 5 |
| 18 | 65 | m | 3 | 32 | 4 | 10 | 18 | 6 | 16 | 2 | 4 |
| 19 | 65 | f | 7.5 | 40 | 13 | 14 | 18 | 15 | 19 | 4 | 1 |
| 20 | 72 | f | 5 | 8 | 14 | 26 | 25 | 34 | 15 | 1 | 3 |
| 21 | 71 | m | 4 | 8 | 6 | 10 | 11 | 15 | 11 | 4 | 2 |
| 22 | 72 | f | 2 | 70 | 0 | 11 | 23 | 15 | NA | NA | NA |
| 23 | 77 | m | 20 | 70 | 10 | 12 | 21 | 38 | 16 | 0 | 2 |
| 24 | 56 | f | 1 | 0.0 | 0 | 11 | 13 | 16 | 15 | 0 | 1 |
| 25 | 65 | f | 2.5 | 60.0 | 19 | 20 | 26 | 28 | 0 | 2 | 0 |
| Legend: Sex is keyed in as m=male and f=female, VAS – visual analogue score (0-100), BDI – Beck’s depression index, S-Anx – State Anxiety, T-Anx – Trait anxiety, PCS – Pain catastrophizing scale, Mc-S – McGill pain questionnaire sensory subscore, Mc-A – McGill pain questionnaire affective subscore, Mc-E – McGill pain questionnaire evaluative subscore, NA – score not available. | | | | | | | | | | | |

| Supplementary table 3. | |
| --- | --- |
| Medicines of osteoarthritis patients | |
| Participant | Medication |
| 1 | tranexamic acid |
| 2 | None taken |
| 3 | None taken |
| 4 | Paracetamol (2 x 500 mg) 3 hours prior to visit |
| 5 | Losartan (25 mg), bendroflumethiazide (2.5 mg), prochlorperazine |
| 6 | None taken |
| 7 | atropine eye drops, maxidex and cosopt eye drops, hypermellose eye drops, latanoprost drops |
| 8 | Paracetamol (1000 mg) 8 hours prior to visit |
| 9 | None taken |
| 10 | Glucosamine (100mg), bendroflumethiazide (2.5mg), simvastatin (20mg), contiflow (400 microg) |
| 11 | Amlodipine (5mg) |
| 12 | Aspirin (75mg-4 hours prior to visit), paracetamol (x8 last 24h, last taken 4 hours prior to visit), simvastatin (40mg) |
| 13 | None taken |
| 14 | Paracetamol (1000mg – taken 2.5 hours prior to visit), aspirin (75mg – taken 4.5 hours prior to visit), co-codamol (2 x 500/30mg – taken 15 hours prior to visit), lansoprazole (20mg), glucosamine (1000mg) |
| 15 | None taken |
| 16 | None taken |
| 17 | None taken |
| 18 | Bendroflumethiazide (2.5 mg), amlodipine (5 mg), ventolin, tiopsol eye drops, predsol eye drops, antibiotic eye drop |
| 19 | Paracetamol (2x500mg – taken 4 hours prior to visit), lansoprazole (30mg), losartan |
| 20 | Ramipril (10mg), Atorvastatin (10mg) |
| 21 | Felodipine (10mg), Ramipril (10mg), Atenolol (100mg), Furosemide (40mg), Warfarin (1.5mg), Candesartan (16mg), Gliclazide (80mg), Doxazosin (4mg), Spironolactone (4mg), Simvastatin |
| 22 | Oxybutynin (10mg) |
| 23 | Paracetamol (1000mg – taken 1 hour prior to study visit), co-codamol (2x30/500mg- taken 16 hours prior to study visit) |
| 24 | None taken |
| 25 | Amlodipine (5mg) |

| Supplementary Table 4 | | | | | | |
| --- | --- | --- | --- | --- | --- | --- |
| Demographics of healthy participants | | | | | | |
| Participant | Age | Sex | BDI | S-Anx | T-Anx | PCS |
| 1 | 59 | F | 0 | 10 | 16 | 7 |
| 2 | 70 | F | 1 | 14 | 23 | 16 |
| 3 | 59 | F | 4 | 10 | 11 | 11 |
| 4 | 65 | M | 1 | 19 | 16 | 18 |
| 5 | 68 | M | 1 | 10 | 21 | 9 |
| 6 | 57 | M | NA | 10 | 11 | 0 |
| 7 | 51 | F | 0 | 10 | 16 | 22 |
| 8 | 66 | F | 1 | 11 | 12 | 5 |
| 9 | 58 | F | 1 | NA | 17 | 7 |
| 10 | 59 | M | 4 | 23 | 14 | 15 |
| 11 | 57 | F | 0 | 12 | 17 | 7 |
| 12 | 55 | F | 0 | 15 | 20 | 13 |
| 13 | 60 | F | 0 | 11 | 18 | 29 |
| 14 | 69 | F | 3 | 10 | 20 | 18 |
| 15 | 72 | M | 1 | 10 | 16 | 17 |
| 16 | 67 | M | 11 | 17 | 23 | 12 |
| 17 | 69 | M | NA | NA | NA | 24 |
| 18 | 70 | M | 3 | 13 | 13 | 8 |
| 19 | 80 | F | 12 | 16 | 21 | 23 |
| Legend: Sex is keyed in as m=male and f=female, BDI – Beck’s depression index, S-Anx – State Anxiety, T-Anx – Trait anxiety, PCS – Pain catastrophizing scale, NA – score not available. | | | | | | |

| Supplementary Table 5 | |
| --- | --- |
| Medications of healthy participants | |
| Participant | Medications |
| 1 | None taken |
| 2 | None taken |
| 3 | None taken |
| 4 | Eye drops for Glaucoma |
| 5 | None taken |
| 6 | None taken |
| 7 | None taken |
| 8 | None taken |
| 9 | None taken |
| 10 | None taken |
| 11 | None taken |
| 12 | None taken |
| 13^#^ | Paracetamol (500 mg - 14.5 hours before visit), losartan (100mg, 4.5 hour prior to visit), bendroflumethiazide (2.5mg, 4.5 hours prior to visit), omeprazole (20mg, 4.5 hours prior to visit) |
| 14 | Fexfenadine (antihistamine, 3 hours prior to visit) |
| 15 | None taken |
| 16 | Simvastatin (40 mg, 18 hours prior to visit), valsartan (40 mg, 18 hours prior to visit), lacidipine (4 mg, 18 hours prior to visit) |
| 17 | ramipril (5 mg), bendroflumethiazide (5 mg), amlodipine (5mg) |
| 18 | Simavastatin (25mg, 15 hours prior to visit) |
| 19 | None taken |
| Legend: Sex is keyed in as m=male and f=female, BDI – Beck’s depression index, S-Anx – State Anxiety, T-Anx – Trait anxiety, PCS – Pain catastrophizing scale, NA – score not available. ^#^This subject reported having had a fall 9 weeks prior to scanning and suffered from a sore shoulder. The paracetamol had been taken prior to sleeping the night before the afternoon scan and so likely had been taken to aid sleeping and had little to no efficacy by time of scanning. | |

References:

[1] Friedman L, Glover GH. Report on a multicenter fMRI quality assurance protocol. Journal of magnetic resonance imaging : JMRI 2006;23(6):827-839.

[2] Jenkinson M, Bannister P, Brady M, Smith S. Improved optimization for the robust and accurate linear registration and motion correction of brain images. NeuroImage 2002;17(2):825-841.
